# Longitudinal patient-reported outcomes and predictors of improvement in chemotherapy-induced peripheral neuropathy a secondary analysis of a randomized trial

**DOI:** 10.3389/fphar.2026.1853626

**Published:** 2026-08-21

**Authors:** Hager Salah, Ahmed Hassan Shaaban, Mona A. Abdelrahman, Hasnaa Osama, Asmaa M. El-Kalaawy

**Affiliations:** 1 Department of Clinical Pharmacy Services, King Hamad University Hospital, Royal Medical Services, Al saya, Bahrain; 2 Department of Clinical Pharmacy, Bahrain Oncology Center, Royal Medical Services, Al saya, Bahrain; 3 Department of Clinical Oncology, Faculty of Medicine, Beni-Suef University, Beni-Suef, Egypt; 4 Department of Clinical Pharmacy, Faculty of Pharmacy, Beni-Suef University, Beni-Suef, Egypt; 5 Department of Pharmacology, Faculty of Medicine, Beni-Suef University, Beni-Suef, Egypt

**Keywords:** Brief Pain Inventory, chemotherapy-induced peripheral neuropathy, duloxetine, EORTC QLQ-CIPN20, FACT/GOG-NTX, Hamilton Anxiety Rating Scale, patient-reported outcomes

## Abstract

**Background:**

Chemotherapy-induced peripheral neuropathy (CIPN) compromises function and quality of life (QoL) and may precipitate chemotherapy modification. We evaluated longitudinal Patient-reported outcome measures (PROMs) during duloxetine-based CIPN management and explored clinical predictors of response.

**Methods:**

This secondary longitudinal analysis was conducted within a randomized clinical cohort receiving duloxetine-based therapy for chemotherapy-induced peripheral neuropathy (CIPN). Of the patients included in the parent trial, 72 with analyzable patient-reported outcome data were included in the present analysis. PROMs were collected at baseline, week 4, and week 8 using the European Organization for Research and Treatment of Cancer Quality of Life Questionnaire–Chemotherapy-Induced Peripheral Neuropathy 20-item module (EORTC QLQ- CIPN20), Functional Assessment of Cancer Therapy/Gynecologic Oncology Group–Neurotoxicity (FACT/GOG-Ntx), Brief Pain Inventory (BPI), and Hamilton Anxiety Rating Scale HAM-A, and were analyzed using generalized estimating equations with multivariable adjustment. Instruments included EORTC QLQ-CIPN20 (0–100; higher = worse), FACT/GOG-Ntx (higher = better), Brief Pain Inventory (BPI) severity and interference (0–10; higher = worse), and. Hamilton Anxiety Rating Scale (HAM-A; higher = worse).

**Results:**

In this PROM analysis, significant improvement was observed across CIPN symptoms, pain, anxiety, and QoL outcomes over 8 weeks. QLQ-CIPN20 total decreased at week 4 (MD −12.64) and week 8 (MD −23.81) versus baseline (both p < 0.001), with sensory (−13.71; −26.45), motor (−10.97; −22.06), and autonomic (−9.00; −18.44) domain reductions (all p < 0.001). FACT/GOG-Ntx increased in week 4 (MD +6.42) and week 8 (MD +7.46) (p < 0.001), with no significant week 4-to-week eight increment. HAM-A decreased (−3.42; −6.77), while BPI severity (−1.55; −2.78) and interference (−1.26; −2.60) improved (all p < 0.001). Higher baseline pain predicted worse CIPN, lower QoL, and higher anxiety; metastatic disease and chemotherapy dose reduction were associated with poorer outcomes; missingness showed no strong baseline predictors.

**Conclusion:**

Duloxetine-based management was associated with rapid, sustained improvement in CIPN symptoms, pain, anxiety, and QoL over 8 weeks, supporting risk-stratified supportive care pathways.

## 1 Introduction

In the last several decades, there have seen a marked improvement in overall and disease-free survival because of ongoing advancements in the early detection and treatment of cancer (Ferlay et al., 2020; Miller et al., 2022; Siegel et al., 2020). The latter is frequently coupled with the use of more rigorous treatment regimens, which highlights long-term negative effects connected to treatment (Rowland and Bellizzi, 2014). Cancers sometimes necessitate the use of specific chemotherapy drugs, which may cause excruciating nerve damage. Chemotherapy-induced peripheral neuropathy (CIPN) can occur in a variety of ways, with the overall dosage and type of medication used having a major impact. In general, between 57.7% and 78.4% of patients report having CIPN symptoms in the month following the end of neurotoxic chemotherapy (Seretny et al., 2014). Patients with this condition primarily experience abnormal sensory perception, which can lead to balance issues and unstable walking in addition to sensations including numbness, burning, tingling, and pain in the hands and feet (Stubblefield et al., 2009). While the symptoms may eventually go away, many individuals often have them for the rest of their lives (Park et al., 2013). Patient-Reported Outcome Measures (PROMs) enable patients to directly communicate their subjective symptoms without relying on clinician interpretation (Basch, 2010). As a result, PROMs are being used increasingly in both clinical and experimental settings to more accurately measure the patient’s experience of CIPN (Calvert et al., 2018; Basch et al., 2018). Doctors often rely on patient-reported symptoms to evaluate neuropathy, although the interpretation of these symptoms might differ significantly among doctors. In research including patients treated with oxaliplatin, 60% of them experienced symptoms of neuropathy. However, doctors only documented 10% of these claimed symptoms (Bennett et al., 2012). The most commonly used scales for patients to report symptoms are the Treatment of Cancer - Quality of Life Questionnaire-Chemotherapy Induced Peripheral Neuropathy (EORTC QLQ-CIPN20) scale (Postma et al., 2005) and the Functional Assessment of Cancer Therapy/Gynecologic Oncology Group Neurotoxicity (FACT/GOG-NTx) questionnaire (Calhoun et al., 2003). Both of these scales employ Likert scales to assess the severity of each symptom, ranging from “not at all” to “very much”. The validity and reliability of these questionnaires have been thoroughly examined (Smith et al., 2013; Kieffer et al., 2017), demonstrating higher sensitivity and consistency in assessing CIPN symptoms compared to the NCI-CTCAE (Smith et al., 2013; Li et al., 2023). While PROMs may not provide information on the underlying physiological processes, they do offer valuable insights into how CIPN symptoms affect patients and help establish a connection between patient experiences and objective CIPN outcomes (Ezendam et al., 2014; Eckhoff et al., 2015). This study is a longitudinal analysis of Patient-reported outcome measures (PROMs) Focusing on participants with analyzable PROM data for the Hamilton Anxiety Rating Scale (HAM-A), FACT/GOG-Ntx, and EORTC QLQ-CIPN20.

## 2 Methodology

### 2.1 Study design and assessment schedule

This secondary analysis was nested within a three-arm randomized trial comparing duloxetine monotherapy, duloxetine plus amitriptyline, and duloxetine plus gabapentin for CIPN management (Salah et al., 2026) by examining patient-reported neuropathy burden, neurotoxicity-related quality of life, and anxiety trajectories in the participants with available PROM data. The present study addresses a different question to evaluate patient-reported outcomes (PROM) over three points: baseline (pre-intervention), week 4, and week 8.

Outcomes were collected using validated PROM instruments capturing chemotherapy-induced peripheral neuropathy (CIPN) symptoms, health-related quality of life (HRQoL), anxiety severity, and pain intensity.

This secondary analysis was nested within a three-arm randomized trial comparing duloxetine monotherapy, duloxetine plus amitriptyline, and duloxetine plus gabapentin for CIPN management. Randomization determined treatment allocation in the parent trial; however, the present PROM analysis was not designed or powered to compare treatment arms. Therefore, participants with analyzable PROM data were pooled to evaluate overall longitudinal patient-reported symptom, pain, anxiety, and quality-of-life trajectories during duloxetine-based management. Treatment augmentation status (none, amitriptyline, or gabapentin) was included in the adjusted GEE models to account for regimen heterogeneity.

### 2.2 Participants and clinical variables

Participants were obtained from the oncology department within a tertiary hospital facility. The experiment occurred from October 2023 until July 2025. Ethical approval (Ref. No. FMBSUREC/03102023) for the study was secured from the Institutional Ethics Committee prior to its commencement.

Among the parent randomized cohort, 72 patients had complete and analyzable patient-reported outcome data and were included in this analysis, as they had complete and analyzable patient-reported outcome data.

Eligible were adults with CTCAE-defined CIPN after exposure to CIPN-inducing chemotherapy and after confirmation of clinician assessment. Clinical variables were recorded to support explanatory modeling and reduce confounding, including (as applicable): sex, metastatic status, number of prior treatment lines, chemotherapy neurotoxicity profile (e.g., taxane, platinum, or combination), neuropathy grade, duloxetine dose, and concomitant neuropathy-directed medications (e.g., gabapentin, amitriptyline, with or without vitamin B complex).

Patients were eligible when they meet the following: must voluntarily sign an informed consent form (ICF) and understand and comply with research requirements, including being 18–75 years old (with cut-offs) on the date of signing. Patients must receive CIPN-inducing chemotherapy. Patients must meet Grade 1 sensory chemotherapy-induced peripheral neuropathy (CIPN) criteria as per NCI Common Toxicity. CTCAE v.5.0 grading system, Eastern Cooperative Oncology Group performance status (ECOG PS): 0–2. While excluded when previous allergic reactions to duloxetine, amitriptyline, or gabapentin; pregnancy; life expectancy less than 6 months; inability or unwillingness to comply with research protocols.

### 2.3 Methods (PROM scoring, validity, and analyses)

#### 2.3.1 Outcomes and assessment schedule

Patient-reported outcome measures (PROMs) were collected at baseline (week 0), week 4, and week 8 to quantify chemotherapy-induced peripheral neuropathy (CIPN) symptom burden, neurotoxicity-related quality of life (QoL), anxiety severity, and pain (severity and interference). The repeated assessment structure was prespecified to capture early response (week 4) and consolidation/maintenance effects (week 8), consistent with prior CIPN supportive-care trials (Smith et al., 2013). Randomization determined treatment allocation in the parent trial; however, the present PROM analysis was not designed or powered to compare treatment arms. Therefore, participants with analyzable PROM data were pooled to evaluate overall longitudinal patient-reported symptom, pain, anxiety, and quality-of-life trajectories during duloxetine-based management. Treatment augmentation status (none, amitriptyline, or gabapentin) was included in the adjusted GEE models to account for regimen heterogeneity.

#### 2.3.2 Instruments, scoring systems, and questionnaire validity

##### 2.3.2.1 EORTC QLQ-CIPN20 (CIPN symptom burden)

CIPN symptoms were measured using the EORTC QLQ-CIPN20, which includes sensory, motor, and autonomic subscales. Items are rated on a 4-point Likert scale (1–4) reflecting symptoms in the prior week. Subscale scores are linearly transformed to a 0–100 scale using the standard EORTC module approach:

Score = 1 − (*RS* − 1)/*range* × 100, where *RS* is the mean of completed items within the subscale and *range = 3* (4–1). Higher scores reflect worse CIPN symptoms (Rattanakrong et al., 2022).

Questionnaire validation (CIPN20): CIPN20 has demonstrated acceptable-to-strong psychometric performance across populations and languages; in a validation study, it showed strong internal consistency overall and expected construct behavior, supporting its use for CIPN symptom assessment (Kieffer et al., 2017; Fayers et al., 2001).

##### 2.3.2.2 FACT/GOG-Ntx (neurotoxicity-related QoL)

FACT/GOG-Ntx provides a QoL-oriented complementary perspective, reflecting neurotoxicity’s impact on daily functioning, wellbeing, and treatment burden. Scores were computed using standard FACIT conventions, where higher scores reflect better QoL. Items are summed per FACIT conventions (including reverse-scoring for negatively phrased items). Higher total scores indicate better HRQoL/fewer neurotoxicity-related problems. Questionnaire validation (FACT-GOG-Ntx): FACT-GOG-Ntx has reported acceptable reliability (e.g., Cronbach’s α > 0.70) and evidence of construct validity (e.g., expected correlations with HRQoL and known-groups differences), supporting its measurement performance in CIPN context (Li et al., 2024).

Missing items were handled using FACIT rules (prorating permitted when sufficient items are completed), which is consistent with common practice in oncology PROM reporting (FACT-GOG-NTX, 2016).

##### 2.3.2.3 Hamilton anxiety rating scale (HAM-A)

HAM-A is a clinician-rated measure of anxiety severity with broad use across medical populations. Total score interpretation followed standard conventions; lower scores indicate improvement (Sperry, 2015).

##### 2.3.2.4 Brief pain inventory (BPI) – severity and interference

Pain severity and interference were measured with BPI subscales; both are responsive to clinical change and widely used in oncology and chronic pain. The BPI demonstrates acceptable internal consistency and a stable two-factor structure (intensity vs. interference), supporting its use for longitudinal pain outcomes (Tan et al., 2004).

### 2.4 Sample size calculation

*A priori* sample size calculation was performed using G*Power (version 3.1), assuming a small-to-moderate within-subject effect (f = 0.20–0.25), α = 0.05, power = 0.80, three repeated measurements, and a correlation of 0.5 between time points. The required sample size was estimated to be 28–42 patients. Ultimately, 72 patients were enrolled, providing substantially higher power for the GEE analyses.

### 2.5 Statistical analysis

Data were analyzed using R software (version 4.4.2; R Foundation for Statistical Computing, Vienna, Austria). Continuous variables were summarized as mean ± standard deviation (SD), while categorical variables were presented as frequencies and percentages.

Longitudinal changes in primary outcomes, quality of life measured by the European Organization for Research and Treatment of Cancer CIPN20 (EORTC CIPN20) and the Functional Assessment of Cancer Therapy–Neurotoxicity (FACT-GOG-Ntx), and anxiety measured by the Hamilton Anxiety Rating Scale–Anxiety subscale (HAM-A) were analyzed using Generalized Estimating Equation (GEE) models with an exchangeable working correlation structure. Mean changes (MC) from baseline and mean differences (MD) from adjusted models were reported with 95% confidence intervals (CI). Pain intensity, assessed by the Brief Pain Inventory–Short Form (BPI-SF), was included as a potential confounder.

To evaluate the robustness of treatment effects while minimizing the risk of overfitting, additional clinically guided multivariable GEE analyses were performed. Covariates were selected *a priori* based on their potential clinical relevance to CIPN severity, disease burden, treatment exposure, and concomitant CIPN-directed management, and included average pain score, CTCAE neuropathy grade, disease stage, chemotherapy duration, CIPN therapy augmentation, and vitamin B12 use. To improve model stability, sparse categories were combined where appropriate (CTCAE Grade 2 and 3; Stage III and IV).

Changes in HAM-A categorical severity levels over time were evaluated using the Stuart–Maxwell test for marginal homogeneity. Pairwise *post hoc* comparisons between baseline, week 4, and week 8 were also performed using the Stuart–Maxwell test with Bonferroni-adjusted p-values.

Although missing data were minimal across the three primary outcomes, MAR testing was still performed to confirm the suitability of the analytical approach. Missingness ranged from 4.2% to 13.9% at follow-up assessments: for EORTC CIPN20, six patients (8.3%) at week 4 and 8 patients (11.1%) at week 8; for FACT- GOG-Ntx, three patients (4.2%) at week 4 and 10 patients (13.9%) at week 8; and for HAM-A, five patients (6.9%) at week 4 and seven patients (9.7%) at week 8. Logistic regression analyses were conducted to evaluate whether missingness was related to baseline demographic, clinical, disease-related, or treatment characteristics (Supplementary Table S1). To improve estimate stability, sparse categories were combined where appropriate, and Firth penalized logistic regression was applied when separation or infinite estimates were encountered. Although a few associations were observed at individual time points, no consistent pattern was identified across outcomes or follow-up visits. Importantly, missingness was not associated with insufficient pain reduction, a key indicator of symptom burden. Collectively, these findings did not provide evidence of a systematic relationship between observed patient characteristics and missingness. While the missingness mechanism cannot be verified empirically, the observed pattern was considered broadly compatible with a Missing at Random (MAR) assumption, supporting the use of Generalized Estimating Equations (GEE) for the analysis of longitudinal changes without imputation.

All statistical tests were two-sided, and p-values <0.05 were considered statistically significant. Significant p-values in tables are formatted in bold italics to aid interpretation.

## 3 Results

### 3.1 Baseline characteristics of patients with chemotherapy-induced peripheral neuropathy

72 contributed analyzable PROM data and comprised the analytic cohort for the present secondary study. Additional follow-up missingness within this subset ranged from 4.2% to 13.S% across the assessed questionnaires.

Table 1 summarizes the baseline characteristics of the 72 patients included in this study, all of whom were diagnosed with chemotherapy-induced peripheral neuropathy (CIPN). The mean age was 49.2 ± 14.3 years, with females representing 58.3% of the cohort. The average BMI was 27.4 ± 6.9 kg/m^2^, and 26.4% of patients reported at least one co-existing disease. Breast cancer was the most common underlying malignancy (47.2%), followed by colorectal cancer (30.6%). Most patients demonstrated stable cancer status at baseline (75.0%) and were in early disease stages (Stage I–III: 97.2%). Around half of the cohort received platinum-based chemotherapy (47.2%), and 79.2% continued treatment for ≥6 months. Surgery was performed in 73.6% of patients, and radiation therapy was received by 36.1%. Approximately one-third of the patients (33.3%) required dose reduction due to treatment-related side effects.

**TABLE 1:** Baseline demographic, clinical, and treatment characteristics of patients with chemotherapy-induced peripheral neuropathy (CIPN) (N = 72).

Variables	Value
Age	49.2 ± 14.3
Gender	
Female	42 (58.3)
Male	30 (41.7)
BMI	27.4 ± 6.9
Co-existing disease	19 (26.4)
Family history	13 (18.1)
Baseline labs	
Serum creatinine	0.8 ± 0.2
Hb at baseline	11.4 ± 1.8
Total leukocyte count	5.7 ± 2.8
Cancer type	
Breast	34 (47.2)
Colo-rectal	22 (30.6)
Others	16 (22.2)
Cancer status	
Progression	11 (15.3)
Regression	7 (9.7)
Stable	54 (75)
Time to progression	
<2 years	6 (8.3)
≥2 years	5 (6.9)
Stable or regressed	61 (84.7)
Metastasis	19 (26.4)
Stage	
I	22 (30.6)
II	23 (31.9)
III	25 (34.7)
IV	2 (2.8)
Chemotherapy class	
Platinum-based	34 (47.2)
Taxane-based	31 (43.1)
Others	7 (9.7)
Chemotherapy total duration	
<6months	15 (20.8)
>6months	57 (79.2)
Dose reduction related to side effect	24 (33.3) surgery
Radiation	26 (36.1)

### 3.2 CIPN severity and management

Table 2 summarizes the severity and treatment characteristics of CIPN at baseline. Most patients had Grade 1 neuropathy (65.3%), while 22.2% and 12.5% presented with Grade 2 and Grade 3 symptoms, respectively, according to CTCAE criteria. The mean baseline pain score, assessed using the Brief Pain Inventory–Short Form (BPI-SF), was 3.1 ± 1.5. All patients were treated with duloxetine, and 72.2% were additionally prescribed either gabapentin (37.5%) or amitriptyline (34.7%). Non-opioid analgesics were frequently used (73.6%), while opioids were prescribed to 30.6% of patients. Vitamin B12 supplementation was reported in 81.9% of the cohort.

**TABLE 2:** Severity and treatment characteristics of chemotherapy-induced peripheral neuropathy (CIPN) among patients (N = 72).

Variables	Value
CIPN characteristics	
CTCAE neuropathy	
Grade 1	47 (65.3)
Grade 2	16 (22.2)
Grade 3	9 (12.5)
BPI-SF average pain score at baseline	3.1 ± 1.5
Other CIPN medications	
Augmentation therapy	
No augmentation	20 (27.8)
Amitriptyline	25 (34.7)
Gabapentin	27 (37.5)
Analgesic use	
Opioid	22 (30.6)
Non opioid	53 (73.6)
B12 use	59 (81.9)

Data are presented as mean ± standard deviation or n (%). CTCAE, common terminology criteria for adverse events; BPI-SF, Brief Pain Inventory–Short Form. All patients received duloxetine; augmentation therapy refers to the use of either gabapentin or amitriptyline. Analgesic classes are not mutually exclusive.

### 3.3 Changes in quality of life and anxiety following duloxetine treatment

Table 3 summarizes the GEE analyses results for the longitudinal outcome changes from baseline through week 8. EORTC CIPN20 total scores were lower at week 4 (MC = −12.47; 95% CI: −16.42 to −8.52; p < 0.001) and week 8 (MC = −24.20; 95% CI: −29.46 to −18.94; p < 0.001) compared with baseline, with a further statistically significant decrease between week 4 and week 8 (MC = −11.73; 95% CI: −15.64 to.

**TABLE 3:** Generalized Estimating Equation (GEE) analyses of changes in CIPN-related quality of life, anxiety, and pain intensity over 8 weeks of duloxetine therapy.

Outcome	M ± SD	MC from baseline (95% CI)	p	MC from week 4 to week 8 (95% CI)	p
EORTC					
Sensory domain baseline	37.3 ± 21.0	References	—	—	—
At week 4	23.7 ± 18.3	−13.71 (−17.62, −9.81	<0.001*	—	—
At week 8	11.3 ± 16.8	−26.45 (−31.59, −21.31)	<0.001*	−12.74 (−16.90, −8.58)	<0.001*
Motor domain baseline	30.7 ± 25.7	References	—	—	—
At week 4	21.0 ± 20.2	−9.99 (−14.66, −5.31)	<0.001*	—	—
At week 8	9.6 ± 16.4	−21.70 (−27.34, −16.06)	<0.001*	−11.71 (−15.80, −7.63)	<0.001*
Autonomic domain baseline	36.9 ± 26.5	References	—	—	—
At week 4	21.8 ± 20.6	−15.07 (−19.33, −10.80)	<0.001*	—	—
At week 8	13.4 ± 21.9	−23.60 (−29.94, −17.25)	<0.001*	−8.53 (−13.23, −3.83)	<0.001*
Total baseline	34.7 ± 22.8	References	—	—	—
At week 4	22.4 ± 18.5	−12.47 (−16.42, −8.52)	<0.001*	—	—
At week 8	0.9 ± 16.7	−24.20 (−29.46, −18.94)	<0.001*	−11.73 (−15.64, −7.83)	<0.001*
FACT-GOG-Ntx PWB					
Baseline	15.0 ± 5.8	Reference	—	—	—
At week 4	18.0 ± 4.4	2.90 (1.65, 4.14)	<0.001*	—	—
At week 8	18.6 ± 5.0	3.70 (2.13, 5.28)	<0.001*	0.81 (−0.09, 1.71)	0.077
SWB baseline	12.6 ± 5.8	Reference	—	—	—
At week 4	15.4 ± 5.2	2.60 (1.34, 3.86)	<0.001*	—	—
At week 8	16.0 ± 5.6	2.98 (1.54, 4.43)	<0.001*	0.39 (−0.47, 1.24)	0.375
EWB baseline	12.9 ± 4.6	Reference	—	—	—
At week 4	15.3 ± 3.5	2.43 (1.51, 3.35)	<0.001*	—	—
At week 8	15.3 ± 3.6	2.60 (1.40, 3.80)	<0.001*	0.17 (−0.59, 0.92)	0.666
FWB baseline	14.2 ± 5.5	Reference	—	—	—
At week 4	16.0 ± 4.8	1.91 (0.57, 3.26)	0.005*	—	—
At week 8	16.9 ± 5.4	2.54 (1.01, 4.07)	0.001*	0.63 (−0.37, 1.62)	0.218
NTX baseline	25.0 ± 9.3	Reference	—	—	—
At week 4	29.3 ± 7.0	4.32 (2.62, 6.02)	<0.001*	—	—
At week 8	29.7 ± 7.6	5.10 (2.80, 7.40)	<0.001*	0.78 (−0.56, 2.11)	0.254
Total baseline	79.6 ± 25.1	Reference	—	—	—
At week 4	94.0 ± 18.1	14.20 (8.99, 19.41)	<0.001*	—	—
At week 8	96.6 ± 21.0	16.94 (10.09, 23.79)	<0.001*	2.74 (−1.06, 6.54)	0.157
HAM-A baseline	29.1 ± 8.7	Reference	—	—	—
At week 4	22.6 ± 7.6	−6.52 (−8.44, −4.60)	<0.001*	—	—
At week 8	20.0 ± 9.4	−9.17 (−11.90, −6.44)	<0.001*	−2.65 (−4.68, −0.61)	0.011*
Average pain scores baseline	3.1 ± 1.5	Reference	—	—	—
At week 4	2.3 ± 1.3	−0.88 (−1.29, −0.46)	<0.001*	—	—
At week 8	1.7 ± 1.3	−1.40 (−1.85, −0.95)	<0.001*	−0.53 (−0.92, −0.13)	0.009*

Data are presented as mean ± standard deviation (M ± SD) or mean change (MC) with 95% confidence intervals (CI). Primary outcomes: CIPN-related quality of life (EORTC CIPN20 and FACT-GOG-Ntx) and anxiety (HAM-A). Pain intensity (BPI-SF) was evaluated as a potential confounder affecting QoL and is reported descriptively. For EORTC CIPN20, lower scores indicate improvement; for FACT-GOG-Ntx, higher scores indicate improvement. GEE, generalized estimating equations; CIPN, Chemotherapy-Induced Peripheral Neuropathy; EORTC CIPN20 = European Organization for Research and Treatment of Cancer CIPN module; FACT-GOG-Ntx, Functional Assessment of Cancer Therapy–Neurotoxicity; PWB, Physical Wellbeing; SWB, Social Wellbeing; EWB, Emotional Wellbeing; FWB, Functional Wellbeing; NTX, neurotoxicity subscale; HAM-A, Hamilton Anxiety Rating Scale–Anxiety Subscale; BPI-SF, Brief Pain Inventory–Short Form.

* p-values <0.05 were considered statistically significant.

−7.83; p < 0.001). FACT-GOG-Ntx total scores were higher at week 4 (MC = 14.20; 95% CI: 8.99 to 19.41; p.

**FIGURE 1:**
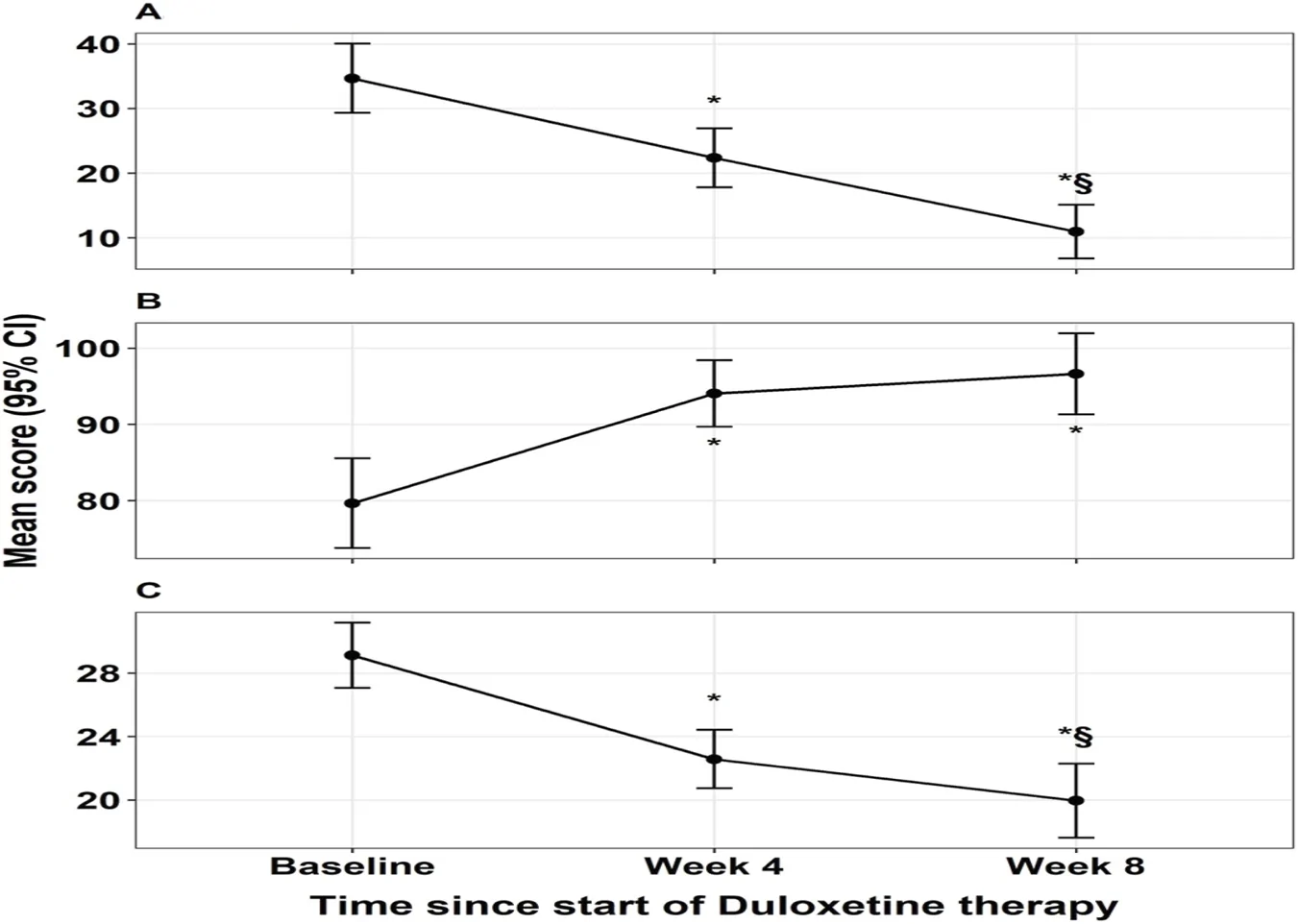
Trends in total CIPN-related quality of life, neurotoxicity, and anxiety scores over 8 weeks of duloxetine therapy in patients with chemotherapy-induced peripheral neuropathy (CIPN). Mean scores (95% CI) of **(A)** total EORTC CIPN20, **(B)** total FACT-GOG-Ntx, and **(C)** HAM-A at baseline, week 4, and week 8 of duloxetine treatment. Improvements are reflected by lower EORTC CIPN20 and HAM-A scores and higher FACT-GOG-Ntx scores. *p < 0.05 vs. baseline. §p < 0.05 vs. week 4. EORTC CIPN20, European Organization for Research and Treatment of Cancer–Chemotherapy-Induced Peripheral Neuropathy module; FACT-GOG-Ntx, Functional Assessment of Cancer Therapy–Neurotoxicity; HAM-A, Hamilton Anxiety Rating Scale–Anxiety subscale.

**FIGURE 2:**
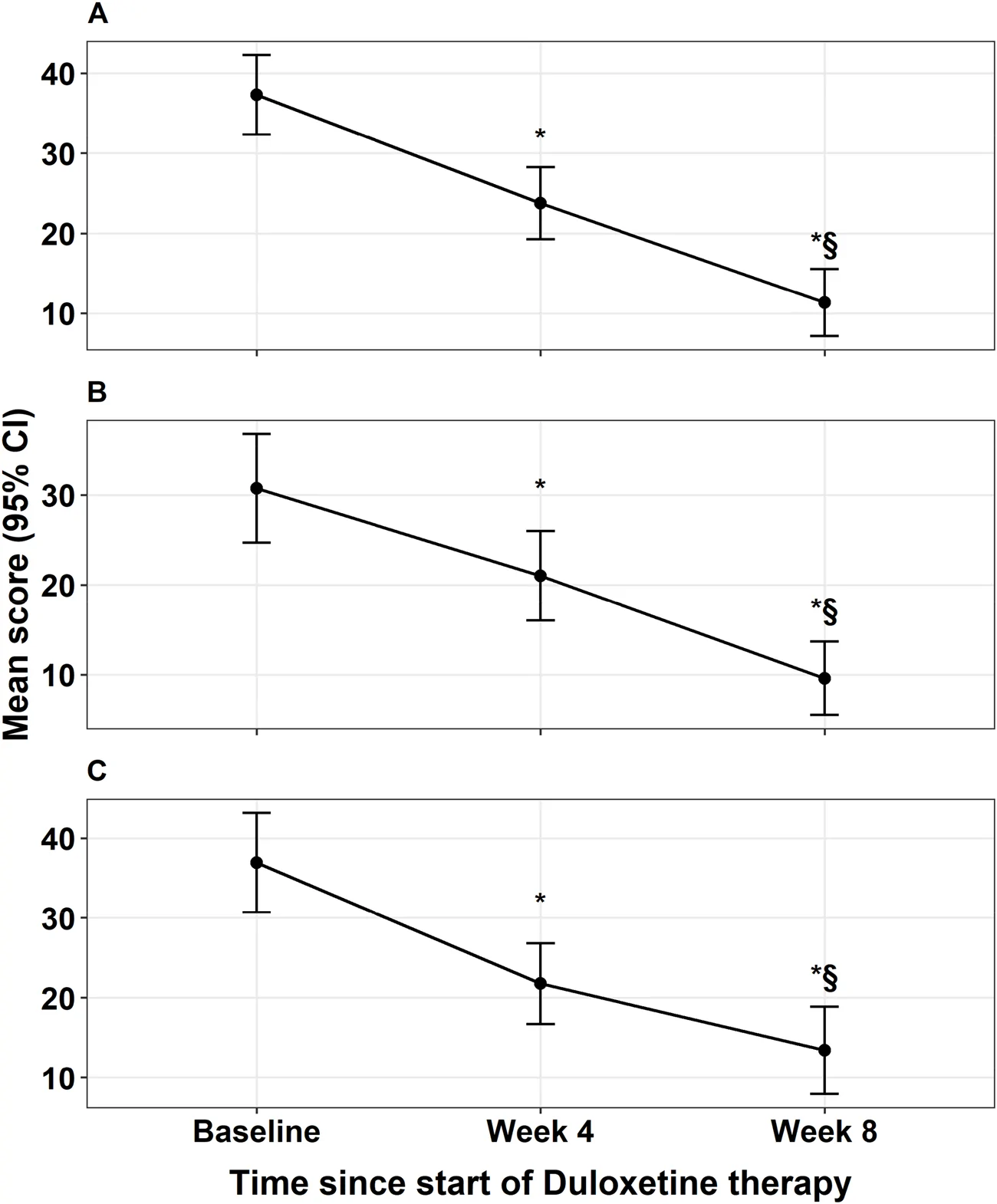
Changes in EORTC CIPN20 sensory, motor, and autonomic domain scores over 8 weeks of duloxetine therapy in patients with chemotherapy-induced peripheral neuropathy (CIPN). Mean scores (95% CI) for the **(A)** sensory, **(B)** motor, and **(C)** autonomic domains of the EORTC CIPN20 at baseline, week 4, and week 8 of duloxetine treatment. Lower scores indicate clinical improvement in CIPN symptoms. *p < 0.05 vs. baseline. §p < 0.05 vs. week 4. EORTC CIPN20, European Organization for Research and Treatment of Cancer–Chemotherapy-Induced Peripheral Neuropathy module.

**FIGURE 3:**
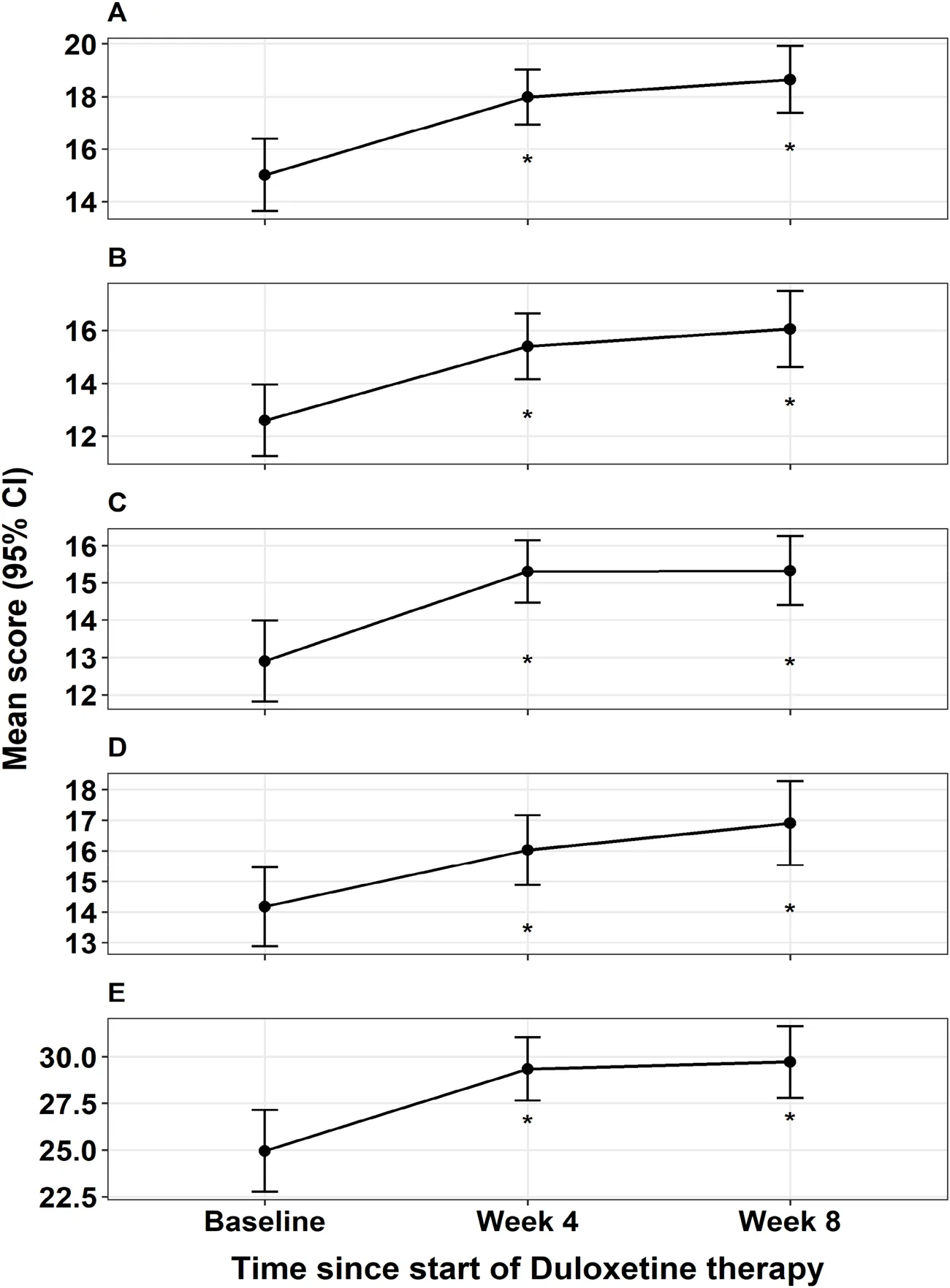
Changes in FACT-GOG-Ntx domain scores over 8 weeks of duloxetine therapy in patients with chemotherapy-induced peripheral neuropathy (CIPN). Mean scores (95% CI) for the **(A)** physical well-being (PWB), **(B)** social well-being (SWB), **(C)** emotional well-being (EWB), **(D)** functional well-being (FWB), and **(E)** neurotoxicity subscale (Ntx) of the FACT-GOG-Ntx at baseline, week 4, and week 8 of duloxetine treatment. Higher scores indicate improvement in health-related quality of life. *p < 0.05 vs. baseline. §p < 0.05 vs. week 4. FACT-GOG-Ntx, Functional Assessment of Cancer Therapy–Neurotoxicity.

< 0.001) and week 8 (MC = 16.94; 95% CI: 10.09 to 23.79; p < 0.001) compared with baseline, while the additional change between week 4 and week 8 was not statistically significant (p = 0.157). The trends in total EORTC CIPN20, FACT-GOG-Ntx, and HAM-A scores are shown in Figure 1. Domain-specific reductions in EORTC sensory, motor, and autonomic subscales are shown in Figure 2. Similarly, increases in the physical, social, emotional, functional, and neurotoxicity subscales of FACT-GOG-Ntx are demonstrated in Figure 3.

HAM-A anxiety scores were lower at week 4 (MC = −6.52; 95% CI: −8.44 to −4.60; p < 0.001) and week 8 (MC = −9.17; 95% CI: −11.90 to −6.44; p < 0.001) compared with baseline, with a further statistically significant decrease from week 4 to week 8 (MC = −2.65; 95% CI: −4.68 to −0.61; p = 0.011).

**FIGURE 4:**
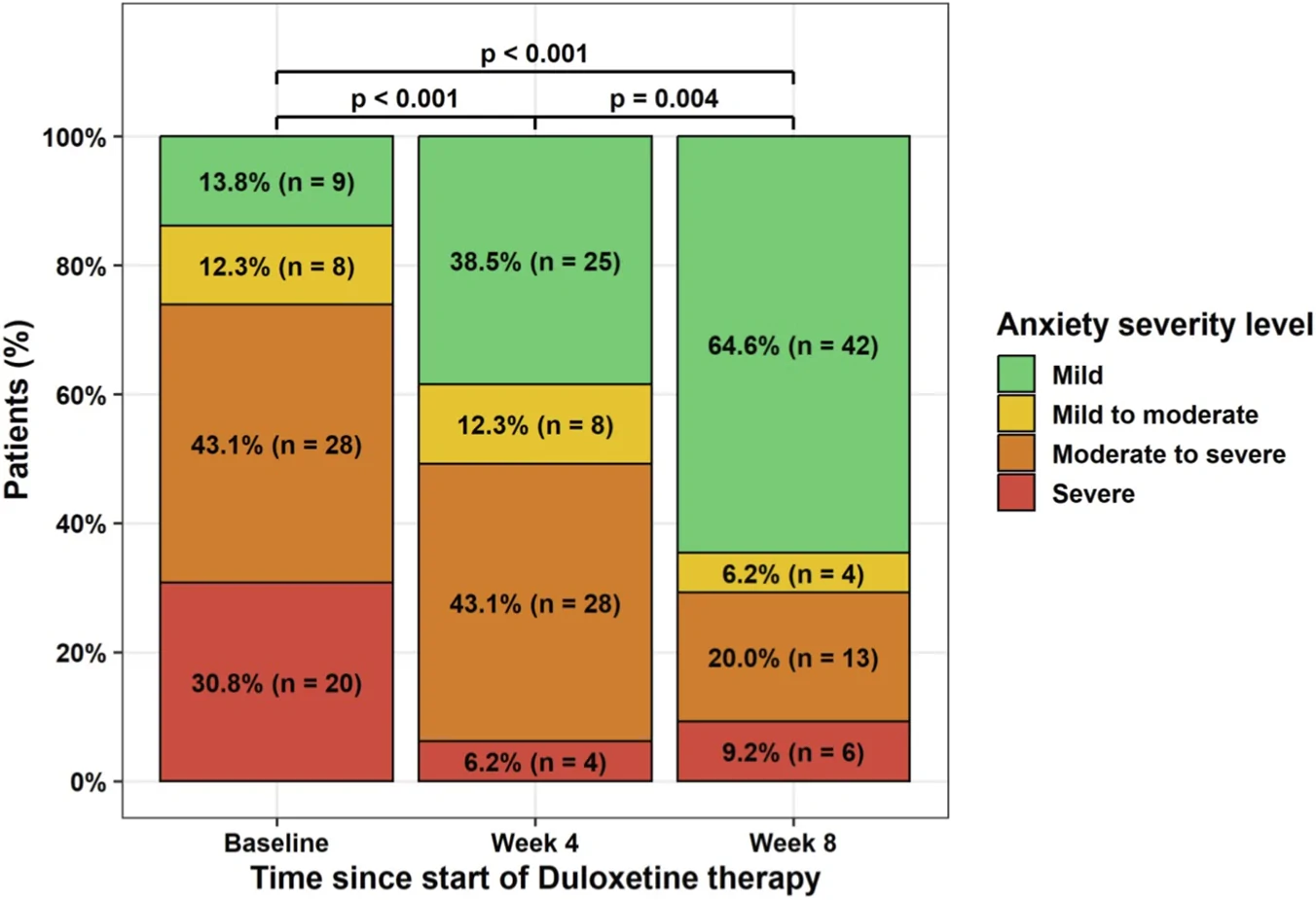
Shifts in anxiety severity levels during 8 weeks of duloxetine therapy based on HAM-A categories. Distribution of anxiety severity levels at baseline, week 4, and week 8 according to the HAM-A A categorical scoring system: mild (green), mild-to-moderate (yellow), moderate-to-severe (orange), and severe (red). Overall and pairwise comparisons across time points were performed using the Stuart–Maxwell test for marginal homogeneity with Bonferroni-adjusted p-values. p < 0.001 for baseline vs. week 4; p < 0.001 for baseline vs. week 8; p = 0.004 for week 4 vs. week 8. HAM-A, Hamilton Anxiety Rating Scale–Anxiety subscale.

Correspondingly, the proportion of patients classified with mild anxiety increased from 13.8% at baseline to 38.5% at week 4% and 64.6% at week 8, while severe anxiety decreased from 30.8% to 6.2% and 9.2%, respectively (p < 0.001 for baseline vs. week 4; p < 0.001 for baseline vs. week 8; p = 0.004 for week 4 vs. week 8) (Figure 4).

Average pain scores (BPI-SF), included as a potential confounder, were also lower at week 4 (MC = −0.88; 95% CI: −1.29 to −0.46; p < 0.001) and week 8 (MC = −1.40; 95% CI: −1.85 to −0.95; p < 0.001) relative to baseline, with a further reduction observed between week 4 and week 8 (MC = −0.53; 95% CI: −0.92 to −0.13; p = 0.009).

### 3.4 Adjusted GEE analyses for factors associated with primary outcomes

Table 4 presents the results of clinically guided multivariable GEE models evaluating factors associated with longitudinal changes in EORTC QLQ-CIPN20, FACT-GOG-Ntx, and HAM-A total scores during 8 weeks of duloxetine-based therapy. After adjustment for average pain score, CTCAE neuropathy grade, disease stage, chemotherapy duration, CIPN therapy augmentation, and vitamin B12 use, time remained significantly associated with improvements across all outcomes. Compared with baseline, EORTC QLQ-CIPN20 total scores were lower at week 4 (MD = −11.38; 95% CI: −15.88 to −6.88; p < 0.001) and week 8 (MD = −22.38; 95% CI: −28.16 to −16.61; p < 0.001). FACT-GOG-Ntx total scores were higher at week 4 (MD = 14.90; 95% CI: 10.02 to 19.78; p < 0.001) and week 8 (MD = 18.24; 95% CI: 11.44 to 25.05; p < 0.001). Similarly, HAM-A total scores were lower at week 4 (MD = −6.19; 95% CI: −8.24 to −4.14; p < 0.001) and week 8 (MD = −8.69; 95% CI: −11.73 to −5.65; p < 0.001) compared with baseline.

**TABLE 4:** Clinically guided multivariable generalized estimating equation (GEE) analyses of factors associated with longitudinal changes in chemotherapy-induced peripheral neuropathy symptoms, neurotoxicity-related quality of life, and anxiety during 8 Weeks of duloxetine-based therapy.

Predictor	EORTC – Total		FACT-GOG-Ntx – Total		HAM-A	
	MD (95% CI)	p	MD (95% CI)	p	MD (95% CI)	p
Time						
Baseline	Reference	—	Reference	—	Reference	—
Week 4	−11.38 (−15.88, −6.88)	***<0.001***	14.90 (10.02, 19.78)	***<0.001***	−6.19 (−8.24, −4.14)	***<0.001***
Week 8	−22.38 (−28.16, −16.61)	***<0.001***	18.24 (11.44, 25.05)	***<0.001***	−8.69 (−11.73, −5.65)	***<0.001***
Average pain score	1.31 (−0.17, 2.80)	0.084	0.84 (−2.60, 4.29)	0.632	0.31 (−0.45, 1.06)	0.429
CTCAE neuropathy grade						
Grade 1	Reference	—	Reference	—	Reference	—
Grade 2–3	−5.30 (−12.36, 1.76)	0.141	1.32 (−5.59, 8.22)	0.708	0.22 (−2.97, 3.42)	0.892
Disease stage						
Stage I–II	Reference	—	Reference	—	Reference	—
Stage III–IV	−2.23 (−9.73, 5.28)	0.561	1.84 (−5.76, 9.45)	0.635	−0.81 (−3.67, 2.05)	0.578
Chemotherapy total duration						
<6 months	Reference	—	Reference	—	Reference	—
>6 months	−15.05 (−25.56, −4.54)	***0.005***	12.32 (3.33, 21.31)	***0.007***	−3.93 (−8.31, 0.45)	0.078
CIPN therapy augmentation						
No augmentation	Reference	—	Reference	—	Reference	—
Amitriptyline	−10.20 (−19.34, −1.05)	***0.029***	20.61 (13.16, 28.05)	***<0.001***	−5.71 (−9.55, −1.87)	***0.004***
Gabapentin	−4.75 (−13.73, 4.23)	0.300	9.00 (0.36, 17.63)	***0.041***	−0.96 (−4.79, 2.87)	0.623
B12 use						
No	Reference	—	Reference	—	Reference	—
Yes	12.06 (1.94, 22.19)	***0.019***	−6.68 (−15.31, 1.96)	0.130	4.00 (0.37, 7.64)	***0.031***

Data are presented as adjusted mean differences (MD) with 95% confidence intervals (CI) obtained from Gaussian generalized estimating equation (GEE) models with an exchangeable working correlation structure and robust standard errors to account for within-patient correlation across repeated assessments. The multivariable models included a limited set of clinically relevant covariates selected *a priori*: time (baseline, week 4, and week 8), average pain score, CTCAE, neuropathy grade (Grade 1 vs. Grade 2–3), disease stage (Stage I–II, vs. Stage III–IV), chemotherapy duration (<6 months vs. ≥ 6 months), CIPN, therapy augmentation (none, amitriptyline, or gabapentin), and vitamin B12 use (yes/no). For EORTC QLQ-CIPN20, and HAM-A, lower scores indicate improvement, whereas for FACT-GOG-Ntx, higher scores indicate improvement. Therefore, negative MD, values for EORTC QLQ-CIPN20, and HAM-A and positive MD, values for FACT-GOG-Ntx reflect more favorable outcomes. Reference categories are indicated in the table. All statistical tests were two-sided, and p-values <0.05 were considered statistically significant. Abbreviations: CIPN, chemotherapy-induced peripheral neuropathy; CTCAE, common terminology criteria for adverse events; EORTC QLQ-CIPN20, European Organization for Research and Treatment of Cancer Quality of Life Questionnaire–Chemotherapy-Induced Peripheral Neuropathy 20-item module; FACT-GOG-Ntx, Functional Assessment of Cancer Therapy/Gynecologic Oncology Group–Neurotoxicity; HAM-A, Hamilton Anxiety Rating Scale–Anxiety subscale; GEE, generalized estimating equation; MD, mean difference; CI, confidence interval. Bold are statistic sig.

Average pain score, CTCAE neuropathy grade, and disease stage were not significantly associated with any of the three outcomes (all p > 0.05). In contrast, chemotherapy duration exceeding 6 months was associated with lower EORTC QLQ-CIPN20 scores (MD = −15.05; 95% CI: −25.56 to −4.54; p = 0.005) and higher FACT-GOG-Ntx scores (MD = 12.32; 95% CI: 3.33 to 21.31; p = 0.007), indicating more favorable neuropathy-related outcomes.

Regarding concomitant CIPN-directed therapy, amitriptyline augmentation was associated with lower EORTC QLQ-CIPN20 scores (MD = −10.20; 95% CI: −19.34 to −1.05; p = 0.029), higher FACT-GOG-Ntx scores (MD = 20.61; 95% CI: 13.16 to 28.05; p < 0.001), and lower HAM-A scores (MD = −5.71; 95% CI: −9.55 to −1.87; p = 0.004) compared with no augmentation. Gabapentin augmentation was associated with higher FACT-GOG-Ntx scores (MD = 9.00; 95% CI: 0.36 to 17.63; p = 0.041), but was not significantly associated with EORTC QLQ-CIPN20 or HAM-A scores. Vitamin B12 use was associated with higher EORTC QLQ-CIPN20 scores (MD = 12.06; 95% CI: 1.94 to 22.19; p = 0.019) and higher HAM-A scores (MD = 4.00; 95% CI: 0.37 to 7.64; p = 0.031), whereas its association with FACT-GOG-Ntx scores did not reach statistical significance (p = 0.130).

## 4 Discussion

This study uniquely integrates neuropathy symptom burden, pain, anxiety, and quality of life within a single longitudinal framework. The present analysis provides complementary insight into longitudinal patient-reported symptom trajectories and predictors. Regarding the overall clinical signal: consistent improvement across CIPN symptoms, QoL, anxiety, and pain Across 8 weeks, there was a coherent and statistically robust improvement in CIPN symptom burden and related patient experience. On EORTC QLQ-CIPN20, all domains improved significantly from baseline to week 4 and further to week 8, with sustained gains between weeks 4 and 8 at sensory, Motor and Autonomic domains with all p < 0.001 and for CIPN20 total: baseline week 4 MD −12.64 (−15.93 to −9.34), baseline week 8 MD −23.81 (−28.13 to −19.49), week 4 and week 8 MD −11.17 (−14.66 to −7.68); all p < 0.001.

These improvements are clinically meaningful because EORTC QLQ-CIPN20 is designed to capture day-to-day neuropathy symptom burden across sensory, motor, and autonomic dimensions, and typically translate to improved functional tolerance of ongoing cancer care (Postma et al., 2005).

The baseline profile of the cohort should be considered as Participants were relatively young and most had CTCAE Grade 1 neuropathy, with a mean baseline BPI-SF average pain score of 3.1 ± 1.5, indicating a predominantly mild pain burden this may limit generalizability to older patients, patients with more severe baseline neuropathy, or patients with established persistent CIPN.

In parallel, QoL improved on FACT/GOG-Ntx overall, though the pattern differed by time window: FACT total improved baseline week 4 MD +6.42 (and baseline week 8 MD +7.46 both p < 0.001, while week 4 to week eight was not significant.

Subdomains showed the same early rise then plateau structure (e.g., physical, social, emotional, functional wellbeing and neurotoxicity subscale all improved significantly from baseline, while most week 4–8 increments were not statistically significant).

Importantly, the magnitude of change observed in our study appears clinically relevant and not merely statistically significant. Published minimal clinically important difference estimates for the EORTC QLQ-CIPN20 sensory and motor domains are approximately 2.5–5.9 and 2.6–5.0 points, respectively, whereas our observed reductions were substantially larger. In addition, Li et al. reported anchor-based minimal important difference estimates of approximately 5.06 points for the QLQ-CIPN20 and 3.54 points for the FACT/GOG-NTX, providing a useful interpretive benchmark for meaningful change. Although those thresholds were derived mainly in cohorts undergoing neurotoxic chemotherapy rather than treatment studies of established CIPN, they support the view that the changes seen in the present cohort likely reflect clinically meaningful improvement (Yeo et al., 2019).

Symptom scores can continue to improve as neuropathic symptom intensity reduces, while broader QoL domains may reach a ceiling or become constrained by other coexisting cancer-related burdens (fatigue, disease anxiety, treatment logistics). Clinically, this underscores why pairing a symptom-specific neuropathy tool (CIPN20) with a QoL-oriented tool (FACT/GOG-Ntx) is informative rather than redundant (Fayers et al., 2001; FACT-GOG-NTX, 2016).

Anxiety and pain improved substantially and in parallel over the 8-week follow-up, with significant reductions in HAM-A, BPI pain severity, and BPI pain interference at both week 4 and week 8, as well as continued improvement between weeks 4 and 8 all p value is significant. This internally consistent pattern suggests that decreasing neuropathic symptom burden was accompanied by less pain intensity, less pain-related disruption of daily functioning, and lower anxiety severity. This is an internally consistent triad response: decreasing neuropathic symptoms aligns with lower pain and interference (BPI), and reduced anxiety severity (HAM-A). The BPI’s psychometric properties support its sensitivity to treatment-related improvement over time, while HAM-A remains a widely accepted anchor for anxiety severity grading in clinical research (Sperry, 2015).

The landmark randomized crossover trial of duloxetine in painful CIPN demonstrated a significantly greater reduction in average pain compared with placebo over 5 weeks, supporting duloxetine’s mechanistic relevance to neuropathic pain modulation. Our observed pain severity reduction by week 8 (MD −2.78 on a 0–10 scale) is not only statistically strong but also consistent with clinically meaningful pain change frameworks that emphasize magnitude and patient-experienced interference (Han and Smith, 2013).

At the same time, the broader duloxetine CIPN evidence base remains mixed when pooled across heterogeneous trials (varying populations, neuropathy phenotypes, timing, and comparators). In 2023 systematic review and meta-analysis reported that duloxetine was statistically similar to placebo across some pooled endpoints, reinforcing the need to interpret benefit in context and to identify response-modifying factors (Chow et al., 2023).

Our multivariable findings add clinically useful nuance by identifying factors independently associated with neuropathy burden (EORTC total), QoL (FACT total), and anxiety (HAM-A): Time effects remained the dominant driver (highly significant at week 4 and week 8 across EORTC total, FACT total, and HAM-A), reinforcing a real longitudinal improvement signal rather than random fluctuation.

Clinically, this supports a pain-anxiety-function linkage: patients entering treatment with greater pain burden are more likely to report worse QoL and anxiety. It also justifies prioritizing baseline pain profiling as part of a CIPN care pathway, because pain appears to be a shared upstream determinant of multiple patient-important outcomes (Tan et al., 2004).

For the findings related to Sex and cancer type: QoL differences are clinically relevant suggest that perceived neurotoxicity burden and recovery are partly shaped by sex-linked symptom appraisal, support systems, and cancer-specific treatment patterns (e.g., regimen neurotoxicity profiles and supportive care intensity). This provides a rationale for stratified supportive care: men and patients with lower baseline QoL might benefit from earlier psychosocial reinforcement and symptom-targeted follow-up (Warrad et al., 2025).

From our study findings we see that Concomitant Amitriptyline use was associated with more favourable outcomes across all three domains: lower EORTC total (MD −16.04; p = 0.010), higher FACT total (MD +24.89; p = 0.007), and lower HAM-A (MD −5.13; p = 0.044). Whereas Gabapentin use was associated with higher FACT total (MD +11.52; p = 0.042).

Because most patients received duloxetine-based combination management, the observed improvements cannot be attributed to duloxetine alone. Although augmentation status was included in adjusted models, associations involving amitriptyline or gabapentin may reflect confounding by indication, clinician assessment, baseline symptom profile, or broader supportive-care intensity.

These associations should not be over-interpreted as causal (confounding by indication is likely), but they are valuable for discussion because the controlled trial literature for gabapentin and amitriptyline in CIPN is inconsistent. For example, a phase 3 crossover trial did not demonstrate benefit for gabapentin in established CIPN symptoms While other smaller prevention-oriented trials have reported benefit in selected settings (e.g., paclitaxel prevention trials. Clinical implications the present findings suggest that duloxetine-centered supportive care may have clinically relevant value in routine CIPN management, particularly for improving symptom burden, pain, anxiety, and patient-reported quality of life over the short term. In practice, this supports maintaining symptom-focused management beyond the early response phase rather than limiting evaluation to the first 4 weeks (Salah et al., 2026). The observed pattern, in which quality-of-life gains were less pronounced after week 4 despite continued symptom improvement, also indicates that pharmacologic treatment alone may be insufficient to sustain broader functional recovery. Accordingly, integration of psychosocial support, rehabilitation, and structured follow-up may be necessary to optimize longer-term patient benefit.

## 5 Limitations

This study has following limitations. The analysis included 72 of 160 randomized patients with analyzable PROM data. The timing of duloxetine initiation relative to chemotherapy and symptom duration or chemotherapy-completion timing were not uniformly available. The follow-up was limited to 8 weeks, so the durability of benefit beyond the short term remains uncertain. The sample was drawn from a single center and was relatively small, which may limit generalizability and reduce precision for subgroup analyses.

## 6 Conclusion

Over 8 weeks, patients with analyzable PROM data demonstrated improvement in CIPN symptom burden, pain, anxiety, and neurotoxicity-related quality of life during duloxetine-based management. These findings support PROM-informed, risk-stratified supportive-care monitoring in patients with painful CIPN, but should be interpreted as short-term longitudinal associations. Further comparative studies with larger, more representative cohorts and longer follow-up are needed to define durability of benefit and identify patients most likely to improve.

## 7 Future directions

Further randomized or comparative trials with extended follow-up are needed to corroborate these findings, evaluate clinically relevant predictors, and define supplementary pharmacologic and non-pharmacologic methods.

## Data Availability

The original contributions presented in the study are included in the article/Supplementary Material, further inquiries can be directed to the corresponding author.
