# Supplementary material for: Longitudinal patient-reported outcomes and predictors of improvement in chemotherapy-induced peripheral neuropathy a secondary analysis of a randomized trial
Source: Front Pharmacol. 2026 Aug 21;17:1853626. doi: 10.3389/fphar.2026.1853626 (PMC13541686; doi:10.3389/fphar.2026.1853626)
Supplement: Supplementary file 1 [file Supplementaryfile1.docx]

| **Supplementary Table S1. Logistic regression analyses assessing predictors of missingness in outcome measures at follow-up assessments** | | | | | | | | | | | | |
| --- | --- | --- | --- | --- | --- | --- | --- | --- | --- | --- | --- | --- |
| **predictor** | **EORTIC** | | | | **FACT-GOG-Ntx** | | | | **HAM-A** | | | |
|  | **At week 4** | | **At week 8** | | **At week 4** | | **At week 8** | | **At week 4** | | **At week 8** | |
|  | **OR**  **(95% CI)** | **p** | **OR**  **(95% CI)** | **p** | **OR**  **(95% CI)** | **p** | **OR**  **(95% CI)** | **p** | **OR**  **(95% CI)** | **p** | **OR**  **(95% CI)** | **p** |
| **Age** | 1.00 (0.94–1.06) | 0.922 | 0.99 (0.94–1.04) | 0.603 | 0.99 (0.91–1.07) | 0.751 | 1.03 (0.98–1.08) | 0.245 | 1.01 (0.94–1.07) | 0.820 | 0.99 (0.94–1.05) | 0.856 |
| **Male Gender** | 0.35 (0.03–1.87) | 0.231 | 0.49 (0.09–2.10) | 0.350 | 0.19 (0.00–2.01) | 0.188 | 0.60 (0.14–2.24) | 0.457 | 0.11 (0.00–1.05) | 0.056 | 0.29 (0.03–1.47) | 0.142 |
| **BMI** | 1.07 (0.94–1.21) | 0.313 | 1.07 (0.96–1.20) | 0.239 | 1.09 (0.91–1.31) | 0.343 | 1.16 (1.03–1.30) | 0.015 | 1.14 (0.98–1.32) | 0.097 | 1.07 (0.95–1.21) | 0.257 |
| **Co-existing disease** | 0.71 (0.07–3.93) | 0.720 | 0.50 (0.05–2.55) | 0.435 | 0.37 (0.00–4.08) | 0.469 | 3.34 (0.88–12.91) | 0.076 | 0.89 (0.09–5.25) | 0.906 | 0.59 (0.06–3.11) | 0.564 |
| **Family history** | 2.50 (0.41–15.38) | 0.323 | 3.24 (0.67–15.77) | 0.145 | 2.37 (0.20–28.36) | 0.494 | 2.23 (0.49–10.11) | 0.299 | 3.39 (0.51–22.75) | 0.208 | 4.12 (0.80–21.30) | 0.091 |
| **Baseline labs** |  |  |  |  |  |  |  |  |  |  |  |  |
| Serum Creatinine | 1.79 (0.01–271.39) | 0.819 | 1.28 (0.02–107.15) | 0.912 | 0.00 (0.00–5.89) | 0.123 | 0.41 (0.01–23.69) | 0.664 | 0.46 (0.00–110.61) | 0.779 | 1.48 (0.01–161.19) | 0.869 |
| Hb at baseline | 1.02 (0.63–1.64) | 0.943 | 1.10 (0.71–1.70) | 0.667 | 0.86 (0.47–1.59) | 0.641 | 1.25 (0.82–1.90) | 0.303 | 0.86 (0.53–1.40) | 0.553 | 0.99 (0.64–1.53) | 0.957 |
| Total Leukocyte Count | 1.04 (0.79–1.36) | 0.796 | 1.19 (0.98–1.46) | 0.085 | 0.75 (0.35–1.58) | 0.444 | 1.06 (0.86–1.31) | 0.576 | 1.01 (0.73–1.38) | 0.971 | 1.01 (0.77–1.32) | 0.945 |
| **Cancer type** |  |  |  |  |  |  |  |  |  |  |  |  |
| Breast | Reference |  | Reference |  | Reference |  | Reference |  | Reference |  | Reference |  |
| Colo-rectal | 0.47 (0.05–2.80) | 0.426 | 0.83 (0.13–4.12) | 0.819 | 0.29 (0.00–3.77) | 0.376 | 0.65 (0.11–3.03) | 0.596 | 0.20 (0.00–2.21) | 0.215 | 0.47 (0.05–2.80) | 0.426 |
| Others | 0.66 (0.06–3.97) | 0.663 | 1.17 (0.19–5.98) | 0.856 | 1.26 (0.11–10.30) | 0.833 | 1.39 (0.29–6.07) | 0.667 | 1.55 (0.24–8.91) | 0.624 | 1.17 (0.19–5.98) | 0.856 |
| **Cancer status** |  |  |  |  |  |  |  |  |  |  |  |  |
| Progression | Reference |  | Reference |  | Reference |  | Reference |  | Reference |  | Reference |  |
| No progression | 0.68 (0.12–7.13) | 0.704 | 0.45 (0.09–2.70) | 0.346 | 0.29 (0.04–3.45) | 0.288 | 1.27 (0.25–12.70) | 0.798 | 0.55 (0.09–5.86) | 0.564 | 0.82 (0.15–8.45) | 0.840 |
| **Cancer stage** |  |  |  |  |  |  |  |  |  |  |  |  |
| Stage I/II | Reference |  | Reference |  | Reference |  | Reference |  | Reference |  | Reference | Reference |
| Stage III/IV | 0.11 (0.00–1.00) | 0.050 | 0.29 (0.03–1.45) | 0.141 | 0.22 (0.00–2.41) | 0.246 | 1.80 (0.48–6.73) | 0.373 | 0.13 (0.00–1.26) | 0.086 | 0.34 (0.03–1.77) | 0.217 |
| **Chemotherapy type** |  |  |  |  |  |  |  |  |  |  |  |  |
| Platinum-Based | Reference |  | Reference |  | Reference |  | Reference |  | Reference |  | Reference |  |
| Non-Platinum -Based | 3.67 (0.68–37.17) | 0.137 | 1.48 (0.36–6.84) | 0.588 | 6.80 (0.63–927.71) | 0.127 | 2.14 (0.58–9.50) | 0.259 | 2.91 (0.50–30.18) | 0.242 | 2.13 (0.48–12.57) | 0.329 |
| **Chemotherapy total Duration** |  |  |  |  |  |  |  |  |  |  |  |  |
| < 6mo | Reference |  | Reference |  | Reference |  | Reference |  | Reference |  | Reference |  |
| >6mo | 3.91 (0.42–520.91) | 0.276 | 5.32 (0.60–702.79) | 0.158 | 1.99 (0.18–273.18) | 0.629 | 1.89 (0.38–18.70) | 0.466 | 3.25 (0.34–435.19) | 0.364 | 4.60 (0.51–610.04) | 0.209 |
| **Dose reduction related to side effect** | 0.50 (0.05–2.74) | 0.452 | 0.73 (0.13–3.13) | 0.680 | 0.27 (0.00–2.90) | 0.317 | 0.90 (0.20–3.38) | 0.881 | 0.16 (0.00–1.52) | 0.127 | 0.42 (0.04–2.16) | 0.320 |
| **Surgery** | 5.34 (0.58–708.53) | 0.163 | 7.29 (0.83–959.22) | 0.079 | 0.60 (0.07–6.85) | 0.639 | 0.76 (0.20–3.44) | 0.702 | 1.12 (0.19–11.73) | 0.906 | 1.69 (0.32–17.00) | 0.564 |
| **Radiation** | 4.00 (0.68–23.55) | 0.125 | 3.41 (0.74–15.66) | 0.114 | 3.75 (0.32–43.50) | 0.291 | 1.21 (0.31–4.76) | 0.783 | 2.87 (0.45–18.41) | 0.266 | 1.37 (0.28–6.66) | 0.697 |
| **CTCEA neuropathy** |  |  |  |  |  |  |  |  |  |  |  |  |
| Grade 1 | Reference |  | Reference |  | Reference |  | Reference |  | Reference |  | Reference |  |
| Grade 2-3 | 1.98 (0.39–10.06) | 0.396 | 2.02 (0.48–8.59) | 0.329 | 14.78 (1.35–2019.56) | 0.025 | 5.15 (1.37–23.25) | 0.015 | 6.49 (1.11–67.66) | 0.037 | 4.88 (1.08–29.06) | 0.040 |
| **Augmentation therapy** |  |  |  |  |  |  |  |  |  |  |  |  |
| No augmentation | Reference |  | Reference |  | Reference |  | Reference |  | Reference |  | Reference |  |
| Any augmentation | 0.69 (0.14–4.22) | 0.658 | 0.58 (0.14–2.72) | 0.467 | 0.22 (0.02–1.73) | 0.144 | 0.51 (0.14–2.06) | 0.332 | 0.25 (0.04–1.38) | 0.109 | 0.46 (0.10–2.26) | 0.228 |
| **Analgesic use** |  |  |  |  |  |  |  |  |  |  |  |  |
| Opioid | 1.15 (0.19–6.80) | 0.877 | 0.73 (0.14–3.95) | 0.718 | 1.14 (0.10–13.31) | 0.915 | 0.97 (0.23–4.16) | 0.967 | 1.57 (0.24–10.11) | 0.637 | 0.90 (0.16–5.04) | 0.905 |
| Non opioid | 5.34 (0.58–708.53) | 0.163 | 0.96 (0.22–5.60) | 0.957 | 0.60 (0.07–6.85) | 0.639 | 1.31 (0.32–7.43) | 0.724 | 1.12 (0.19–11.73) | 0.906 | 0.79 (0.17–4.74) | 0.779 |
| **B12 use** | 3.28 (0.35–437.67) | 0.355 | 1.19 (0.23–12.02) | 0.852 | 0.36 (0.04–4.21) | 0.370 | 0.43 (0.11–2.00) | 0.263 | 0.68 (0.11–7.17) | 0.701 | 0.46 (0.10–2.84) | 0.373 |
| **Insufficient pain reduction (<30% reduction in average pain score)** |  |  |  |  |  |  |  |  |  |  |  |  |
| At week 4 | 0.24 (0.03–2.17) | 0.204 | 0.40 (0.08–2.15) | 0.286 | 2.76 (0.24–31.89) | 0.416 | 0.52 (0.12–2.20) | 0.375 | 0.87 (0.14–5.57) | 0.886 | 0.99 (0.21–4.79) | 0.991 |
| At week 8 |  |  | 1.42 (0.31–6.55) | 0.652 |  |  | 0.97 (0.23–4.16) | 0.967 |  |  | 3.48 (0.71–17.12) | 0.125 |

Values represent odds ratios (OR) with 95% confidence intervals (CI) for the association between baseline demographic, clinical, disease-related, and treatment variables and the occurrence of missing data in total scores of EORTC CIPN20, FACT-GOG-Ntx, and HAMA-A at week 4 and week 8. Missingness proportions at each assessment were: EORTC CIPN20 — 6 (8.3%) at week 4 and 8 (11.1%) at week 8; FACT-GOG-Ntx — 3 (4.2%) at week 4 and 10 (13.9%) at week 8; HAMA-A — 5 (6.9%) at week 4 and 7 (9.7%) at week 8. P-values < 0.05 were considered statistically significant.

| **Supplementary Table S2.** | | | | |
| --- | --- | --- | --- | --- |
| **Variables** | **Total (N = 160)** | **No PROM available**  **(n = 88)** | **PROM available (n = 72)** | **p** |
| Age | 48.9 ± 13.6 | 48.7 ± 13.1 | 49.2 ± 14.3 | 0.818 |
| Gender |  |  |  |  |
| Female | 99 (61.9) | 57 (64.8) | 42 (58.3) | 0.404 |
| Male | 61 (38.1) | 31 (35.2) | 30 (41.7) |  |
| BMI | 28.3 ± 6.7 | 29 ± 6.5 | 27.4 ± 6.9 | 0.145 |
| Serum Creatinine | 0.8 ± 0.2 | 0.8 ± 0.2 | 0.8 ± 0.2 | 0.086 |
| Hb at baseline | 11.7 ± 1.8 | 11.9 ± 1.8 | 11.4 ± 1.8 | 0.133 |
| Total Leukocyte Count | 5.7 ± 3 | 5.8 ± 3.2 | 5.7 ± 2.8 | 0.932 |
| Treatment group |  |  |  |  |
| Duloxetine monotherapy | 47 (29.4) | 27 (30.7) | 20 (27.8) | 0.752 |
| Duloxetine + Amitriptyline | 58 (36.2) | 33 (37.5) | 25 (34.7) |  |
| Duloxetine + Gabapentin | 55 (34.4) | 28 (31.8) | 27 (37.5) |  |
| CTCEA neuropathy |  |  |  |  |
| Grade 1 | 109 (68.1) | 62 (70.5) | 47 (65.3) | 0.468 |
| Grade 2 | 29 (18.1) | 13 (14.8) | 16 (22.2) |  |
| Grade 3 | 22 (13.8) | 13 (14.8) | 9 (12.5) |  |
| Opioid analgesic use | 69 (43.1) | 47 (53.4) | 22 (30.6) | ***0.004**** |
| Non opioid analgesic use | 117 (73.1) | 64 (72.7) | 53 (73.6) | 0.9 |
| Family history | 35 (21.9) | 22 (25) | 13 (18.1) | 0.29 |
| B12 use | 128 (80) | 69 (78.4) | 59 (81.9) | 0.578 |
| Co-existing disease | 32 (20) | 13 (14.8) | 19 (26.4) | 0.068 |
| Cancer type |  |  |  |  |
| Breast | 76 (47.5) | 42 (47.7) | 34 (47.2) | 0.769 |
| Colo-rectal | 45 (28.1) | 23 (26.1) | 22 (30.6) |  |
| Others | 39 (24.4) | 23 (26.1) | 16 (22.2) |  |
| Cancer status |  |  |  |  |
| Progression | 21 (13.1) | 10 (11.4) | 11 (15.3) | 0.745 |
| Regression | 17 (10.6) | 10 (11.4) | 7 (9.7) |  |
| Stable | 122 (76.2) | 68 (77.3) | 54 (75) |  |
| Time to progression |  |  |  |  |
| < 2 years | 11 (6.9) | 5 (5.7) | 6 (8.3) | 0.726 |
| ≥2 years | 10 (6.2) | 5 (5.7) | 5 (6.9) |  |
| stable_or_regressed | 139 (86.9) | 78 (88.6) | 61 (84.7) |  |
| Stage |  |  |  |  |
| Stage I | 57 (35.6) | 35 (39.8) | 22 (30.6) | 0.107 |
| Stage II | 40 (25) | 17 (19.3) | 23 (31.9) |  |
| Stage III | 53 (33.1) | 28 (31.8) | 25 (34.7) |  |
| Stage IV | 10 (6.2) | 8 (9.1) | 2 (2.8) |  |
| Metastasis | 49 (30.6) | 30 (34.1) | 19 (26.4) | 0.293 |
| Treatment start with chemotherapy | 122 (76.2) | 71 (80.7) | 51 (70.8) | 0.145 |
| Chemotherapy class |  |  |  |  |
| Platinum-Based Chemotherapy | 76 (47.5) | 42 (47.7) | 34 (47.2) | 0.989 |
| Taxane-Based Chemotherapy (Taxol-Dominant) | 68 (42.5) | 37 (42) | 31 (43.1) |  |
| Others | 16 (10) | 9 (10.2) | 7 (9.7) |  |
| Chemotherapy total Duration |  |  |  |  |
| <6mo | 35 (21.9) | 20 (22.7) | 15 (20.8) | 0.773 |
| >6mo | 125 (78.1) | 68 (77.3) | 57 (79.2) |  |
| Dose reduction related to side effect | 53 (33.1) | 29 (33) | 24 (33.3) | 0.96 |
| Surgery | 113 (70.6) | 60 (68.2) | 53 (73.6) | 0.453 |
| Radiation | 70 (43.8) | 44 (50) | 26 (36.1) | 0.078 |
